# Supplementary material for: Novel Acetamide-Based HO-1 Inhibitor Counteracts Glioblastoma Progression by Interfering with the Hypoxic–Angiogenic Pathway
Source: Int J Mol Sci. 2024 May 15;25(10):5389. doi: 10.3390/ijms25105389 (PMC11121434; doi:10.3390/ijms25105389)
Supplement: Supplementary file 1 [file ijms-25-05389-s001.zip › Supplementary files/Supplementary file (S3).pdf]

Figure 3 - Panel A

(from 1 to 10)

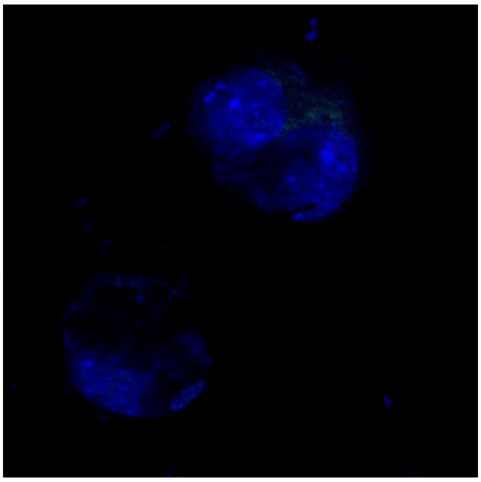

1

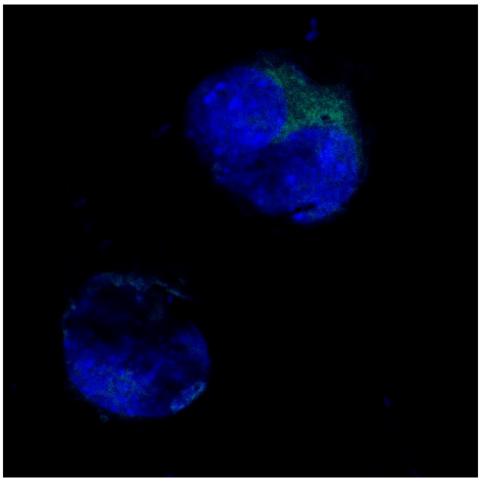

2

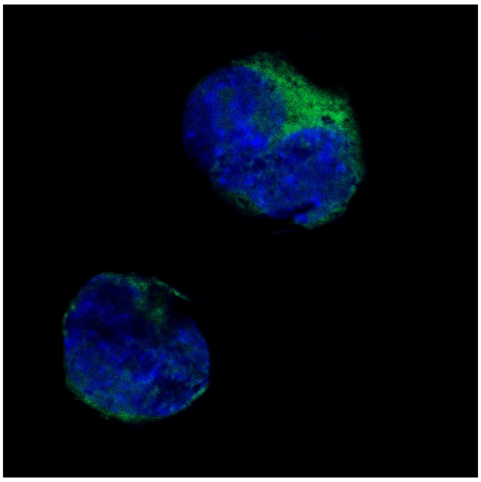

3

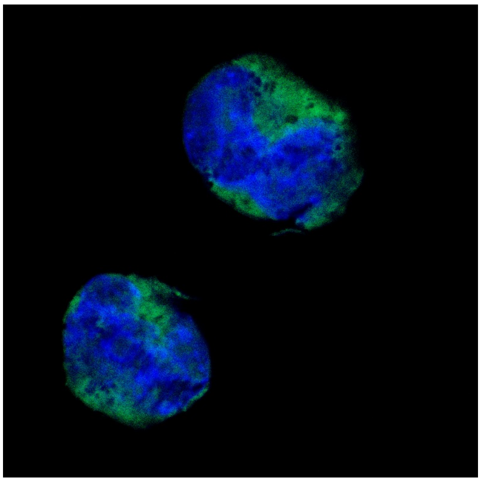

4

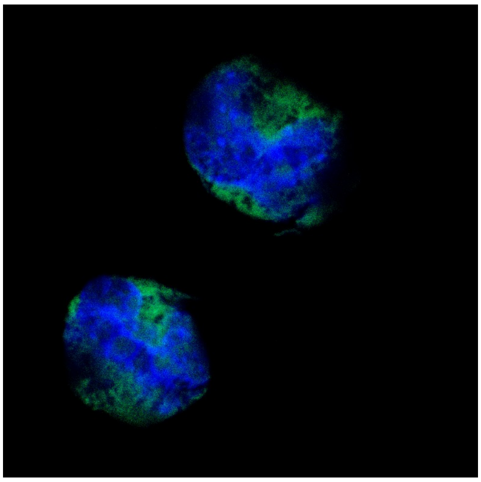
5

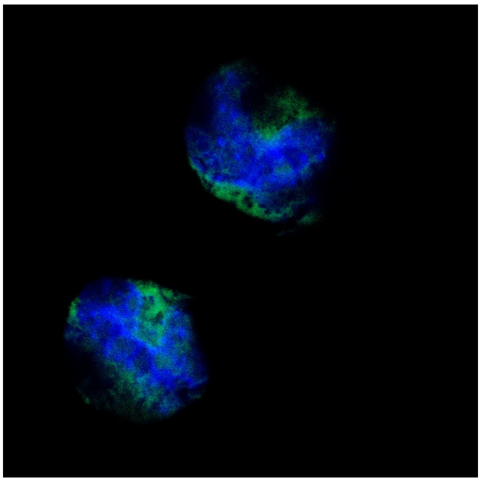
6

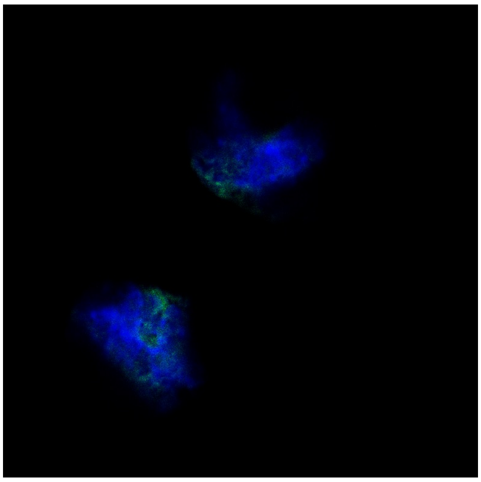
7

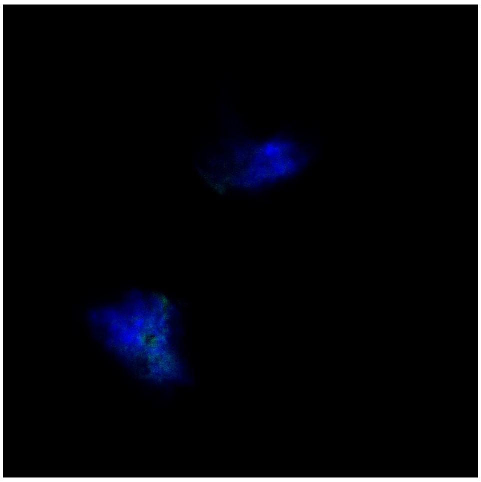
8

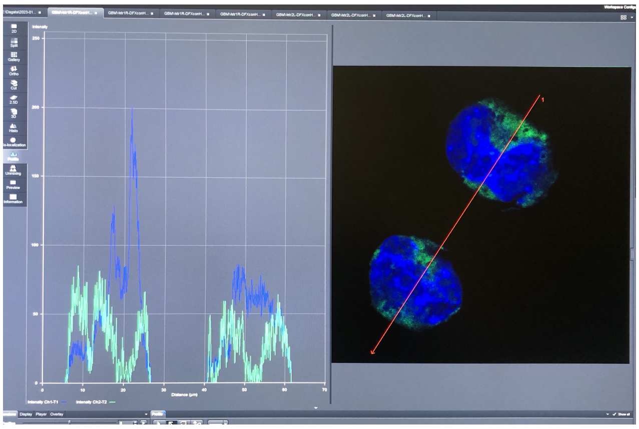

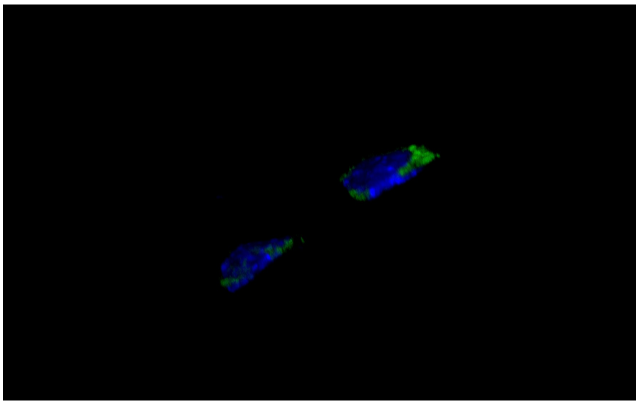

9

10

11

12

Figure 3 - Panel B

(from 13 to 22)

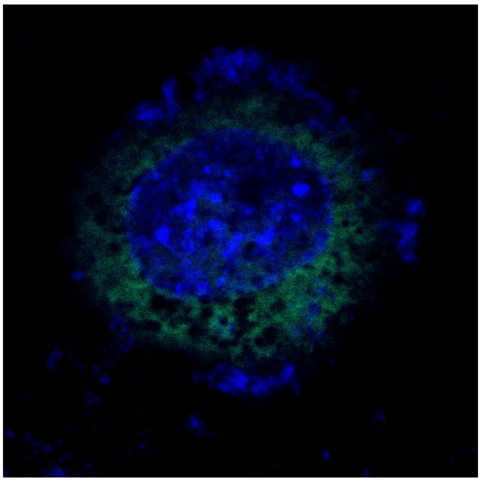

13

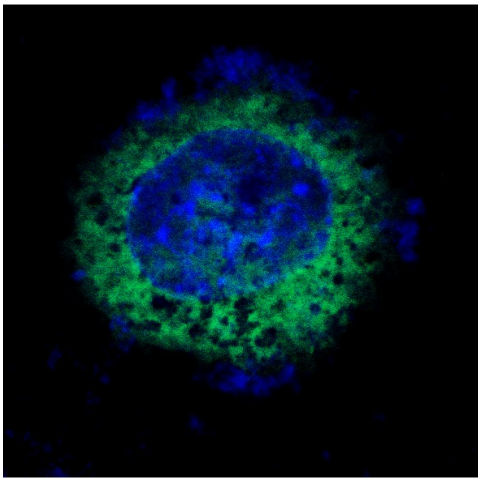

14

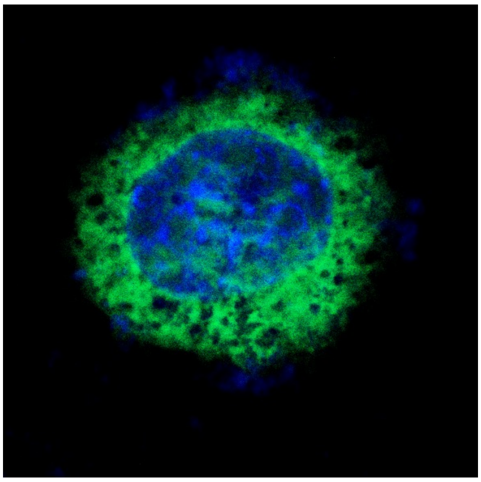

15

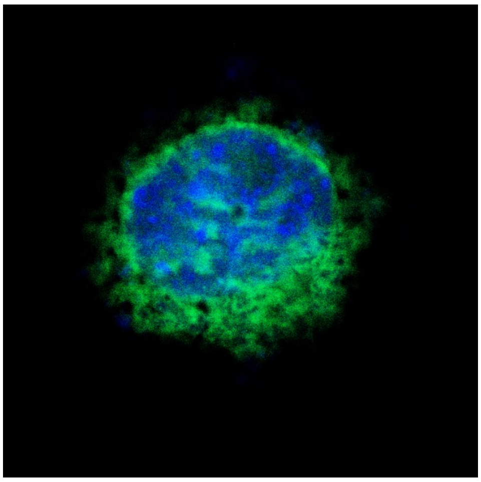

16

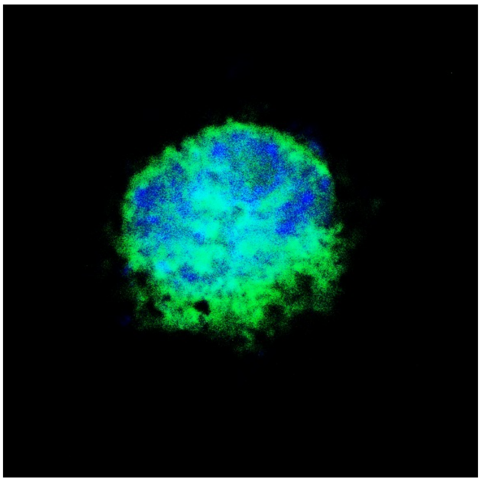

17

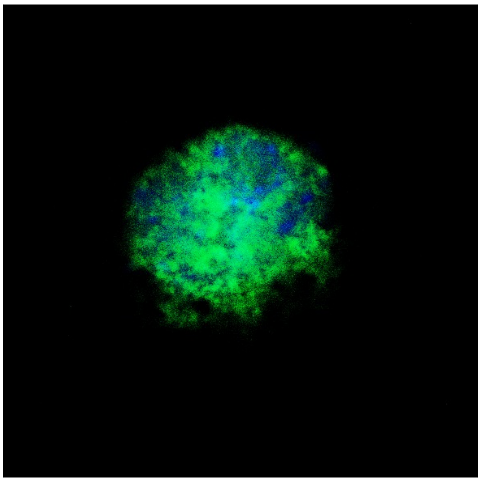

18

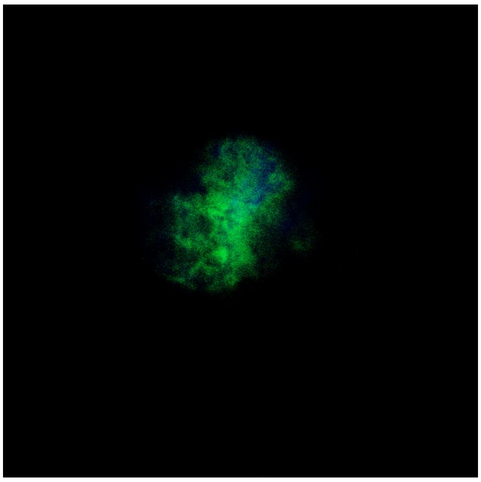

19

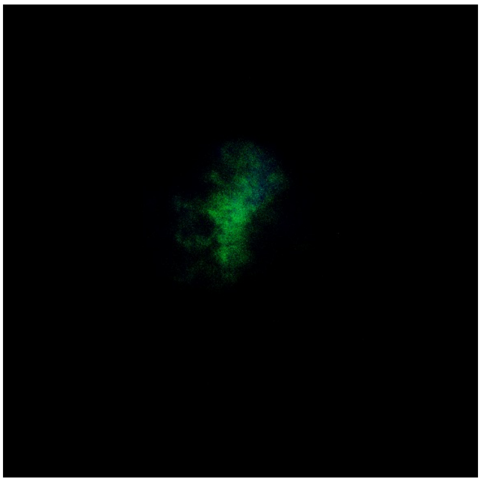

20

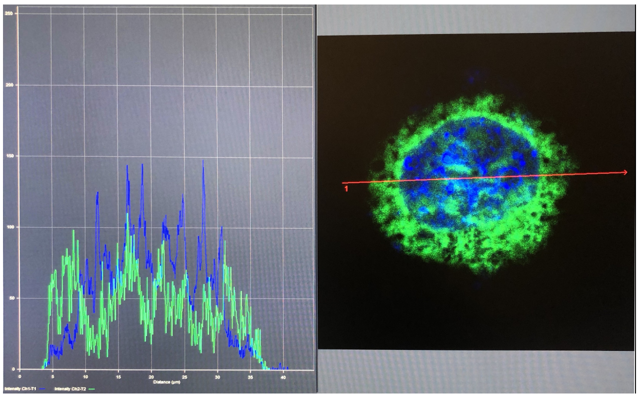
50
0
0 5 10 15 20 25 30 35 40
Distance (µm)
Intensity Ch1-T1
Intensity Ch2-T2
1

21

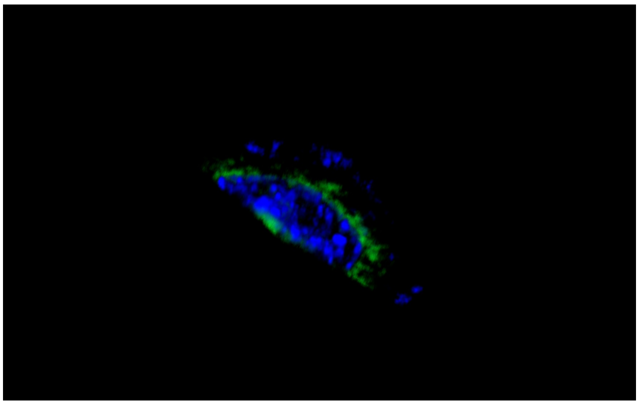

22

23

24

Figure 3 - Panel C

(from 25 to 34)

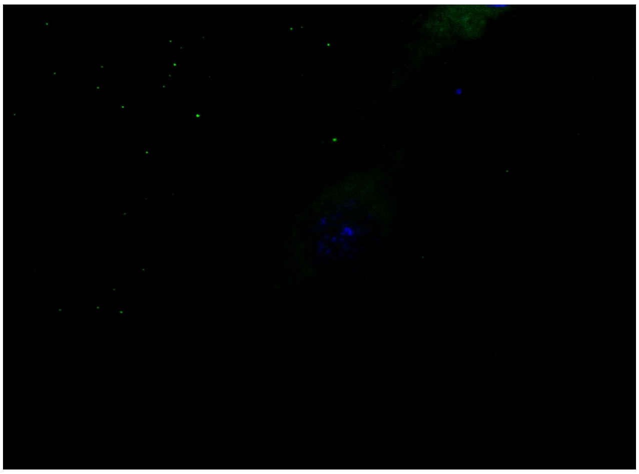

25

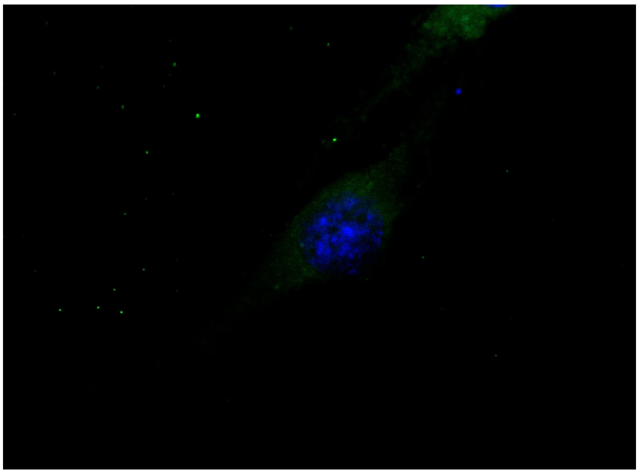

26

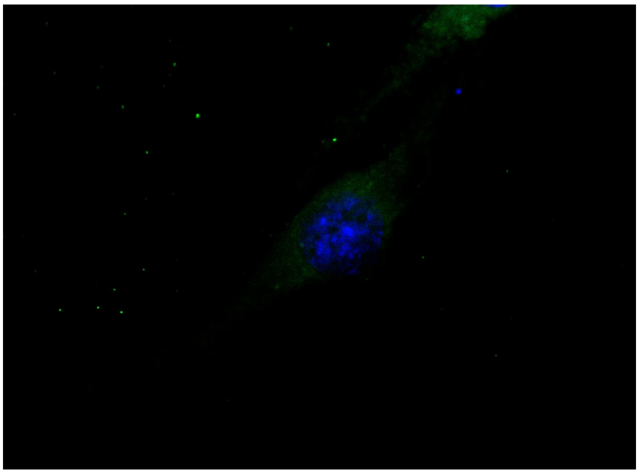

27

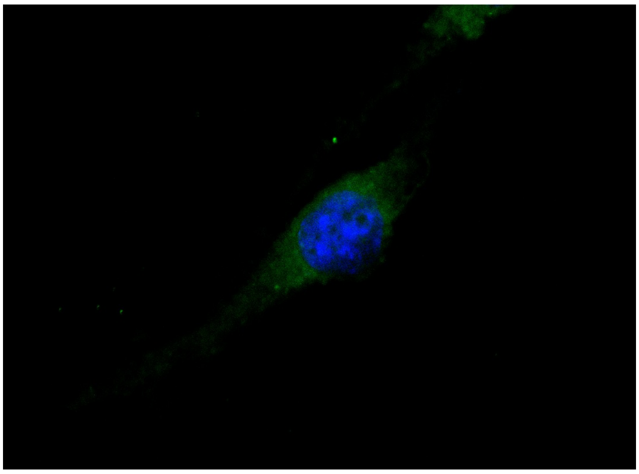

28

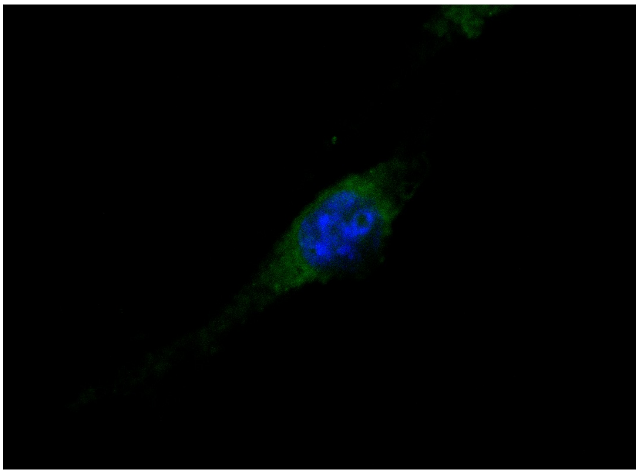
29

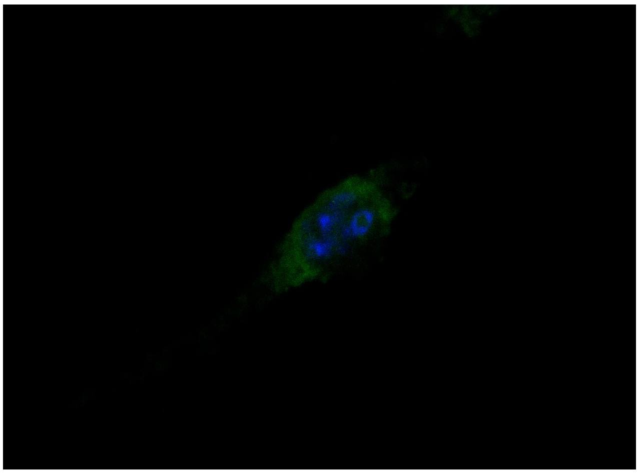
30

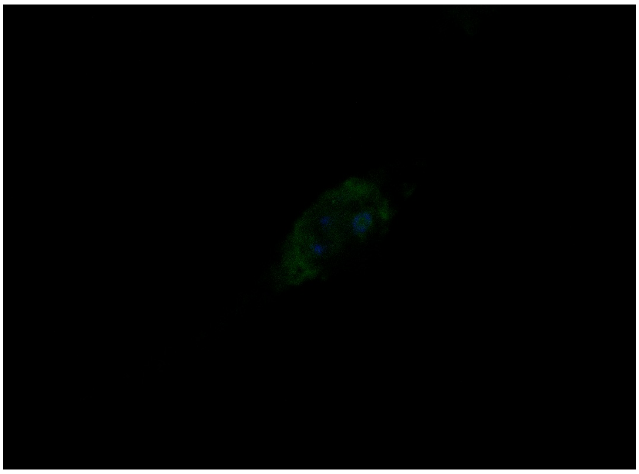
31

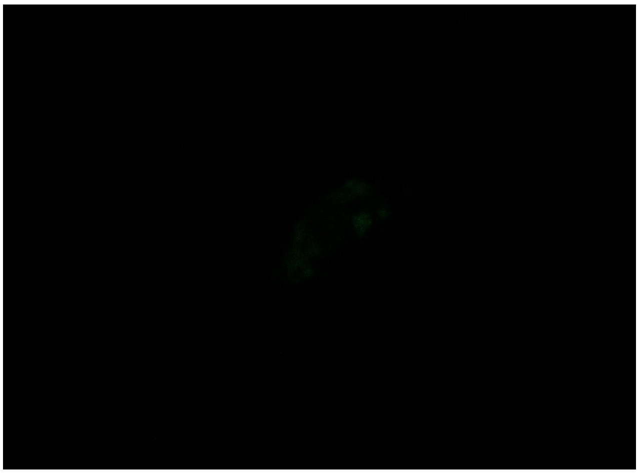
32

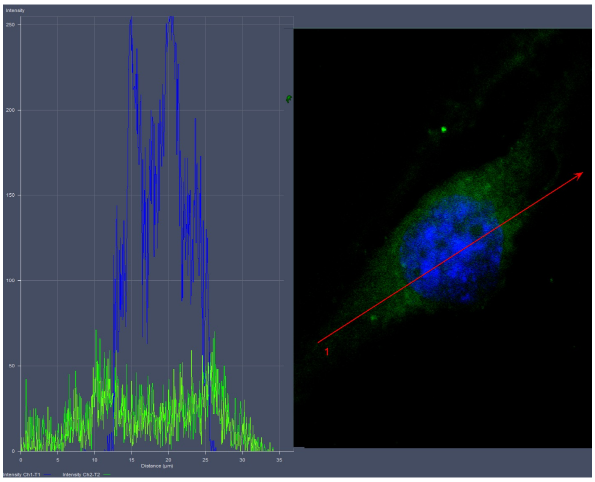
Intensity
250
200
150
100
50
0
0
5
10
15
20
25
30
35
Distance (µm)
Intensity Ch1-T1
Intensity Ch2-T2
1

33

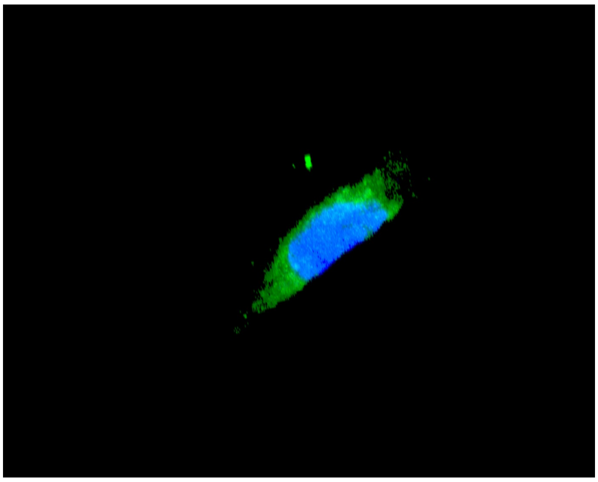

34

35

36

Figure 3 - Panel D

(from 37 to 46)

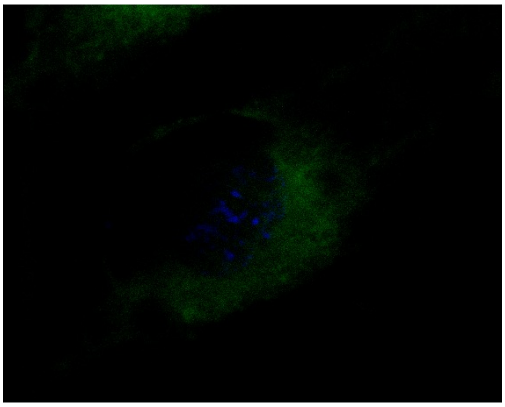

37

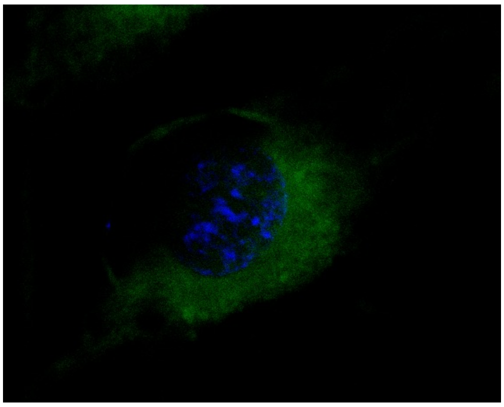

38

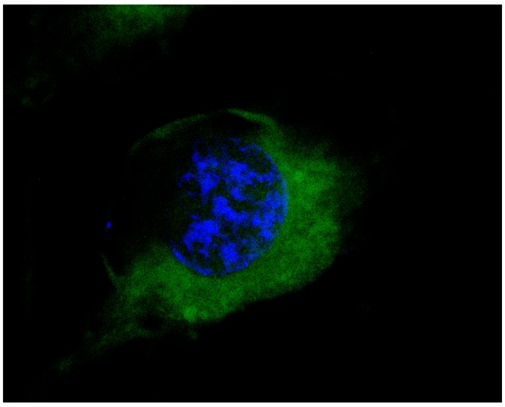

39

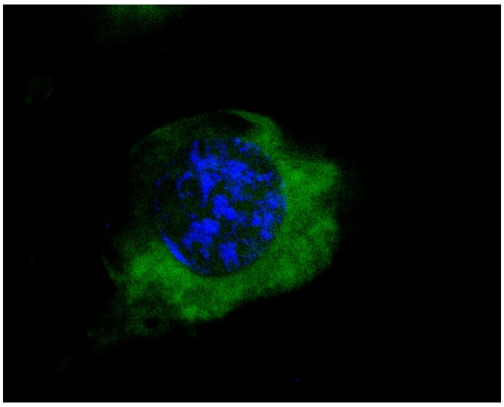

40

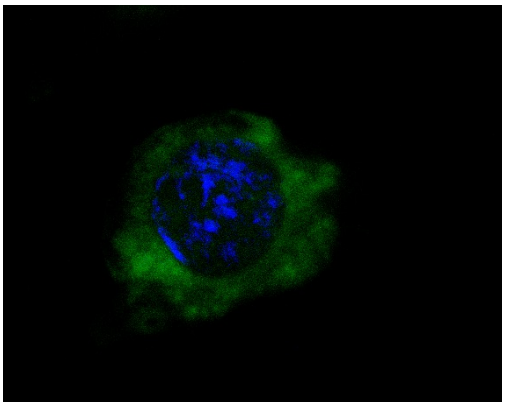

41

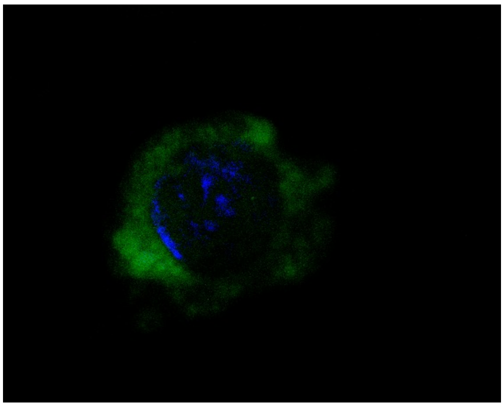

42

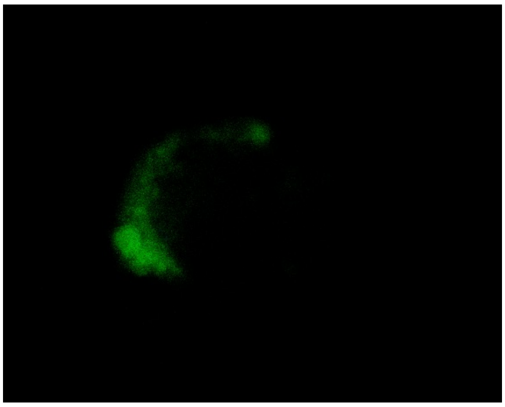

43

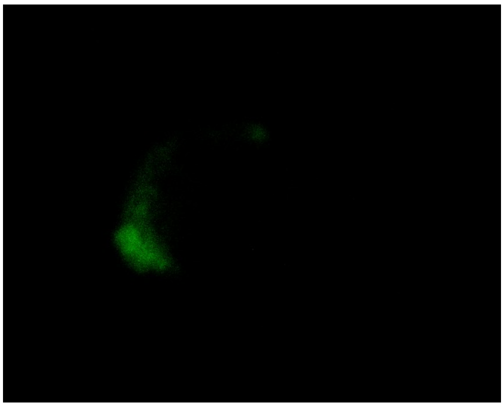

44

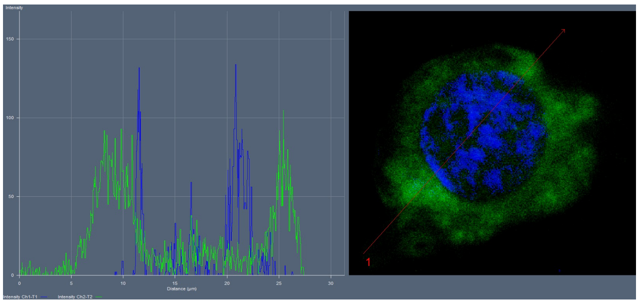
Intensity
150
100
50
0
0
5
10
15
20
25
30
Distance (µm)
Intensity Ch1-T1
Intensity Ch2-T2
1

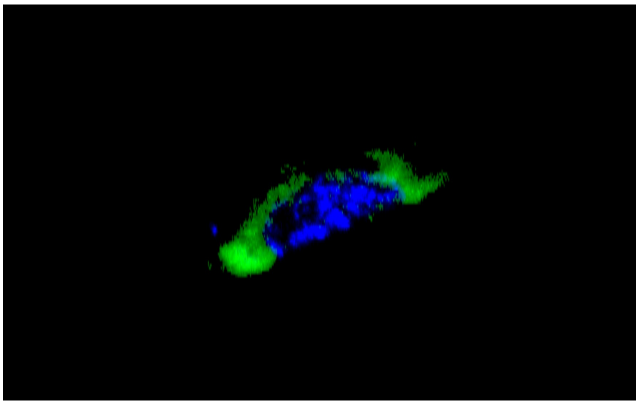

45

46

47

48

Figure 7 - Panel A

(from 49 to 52)

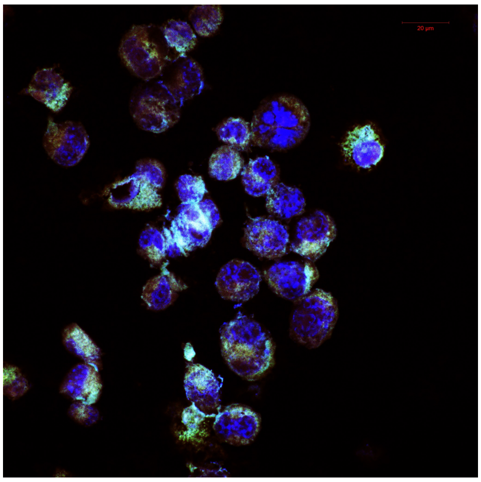

49

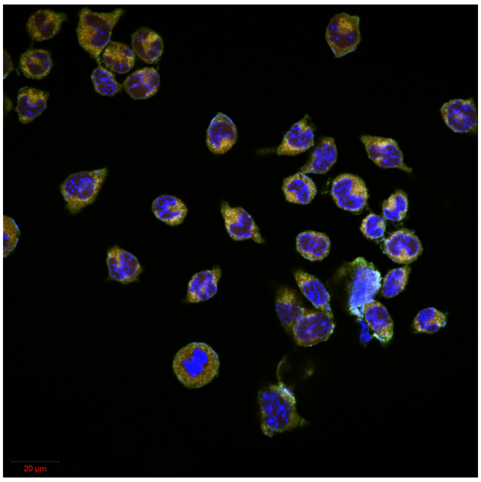

50

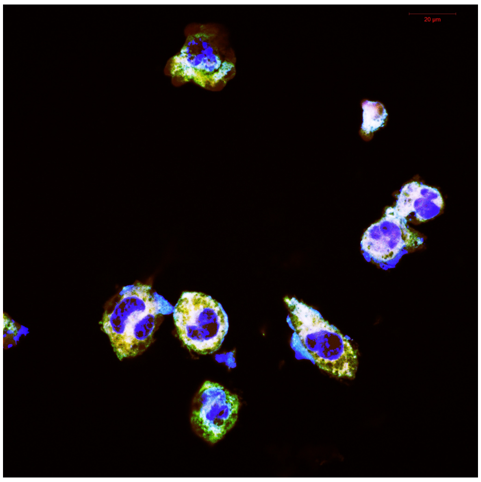

51

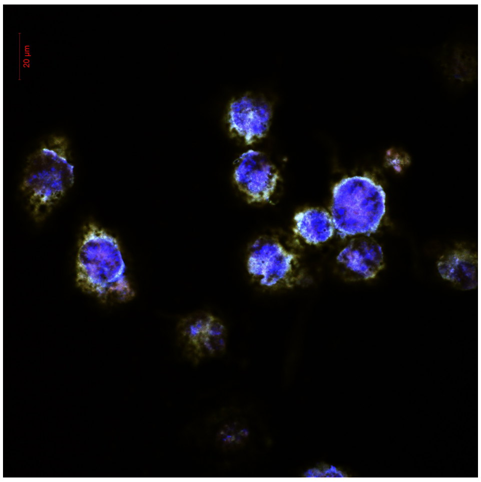

52

Figure 7 - Panel B

(from 53 to 56)

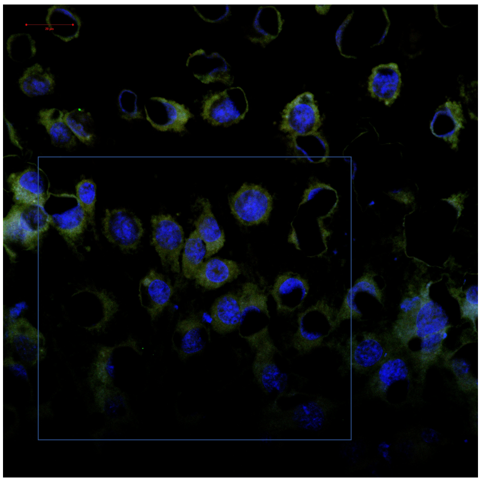

53

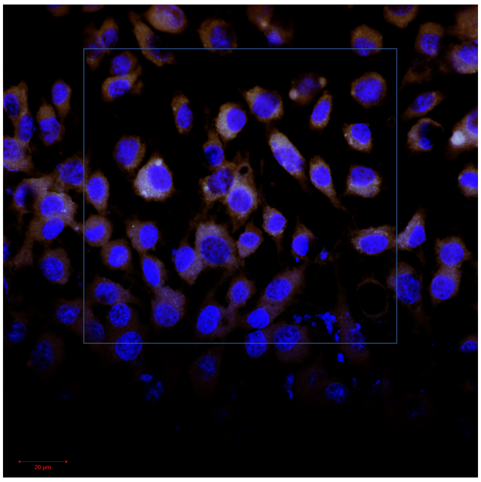

54

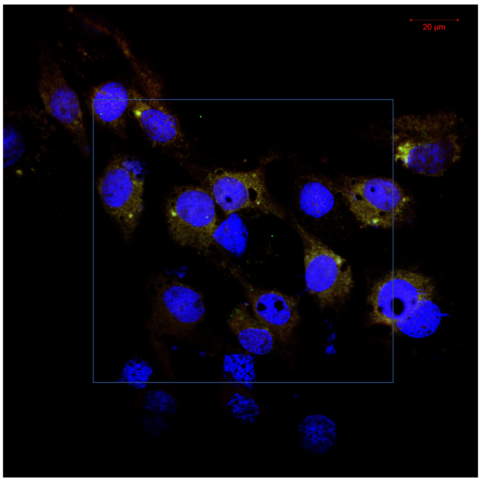

55

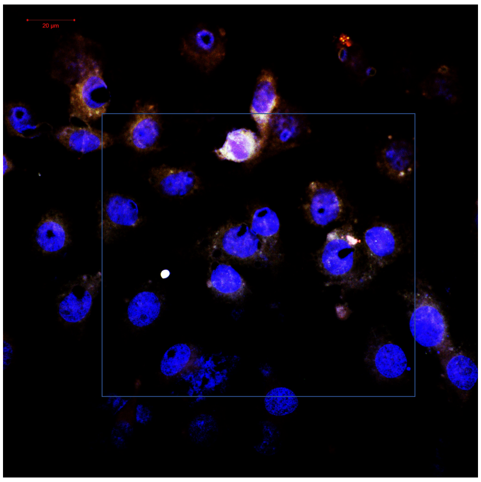

56
