# Supplementary figures and images for: Novel Acetamide-Based HO-1 Inhibitor Counteracts Glioblastoma Progression by Interfering with the Hypoxic–Angiogenic Pathway
Source: Int J Mol Sci. 2024 May 15;25(10):5389. doi: 10.3390/ijms25105389 (PMC11121434; doi:10.3390/ijms25105389)

**Figure 8 A)**

**0 h**

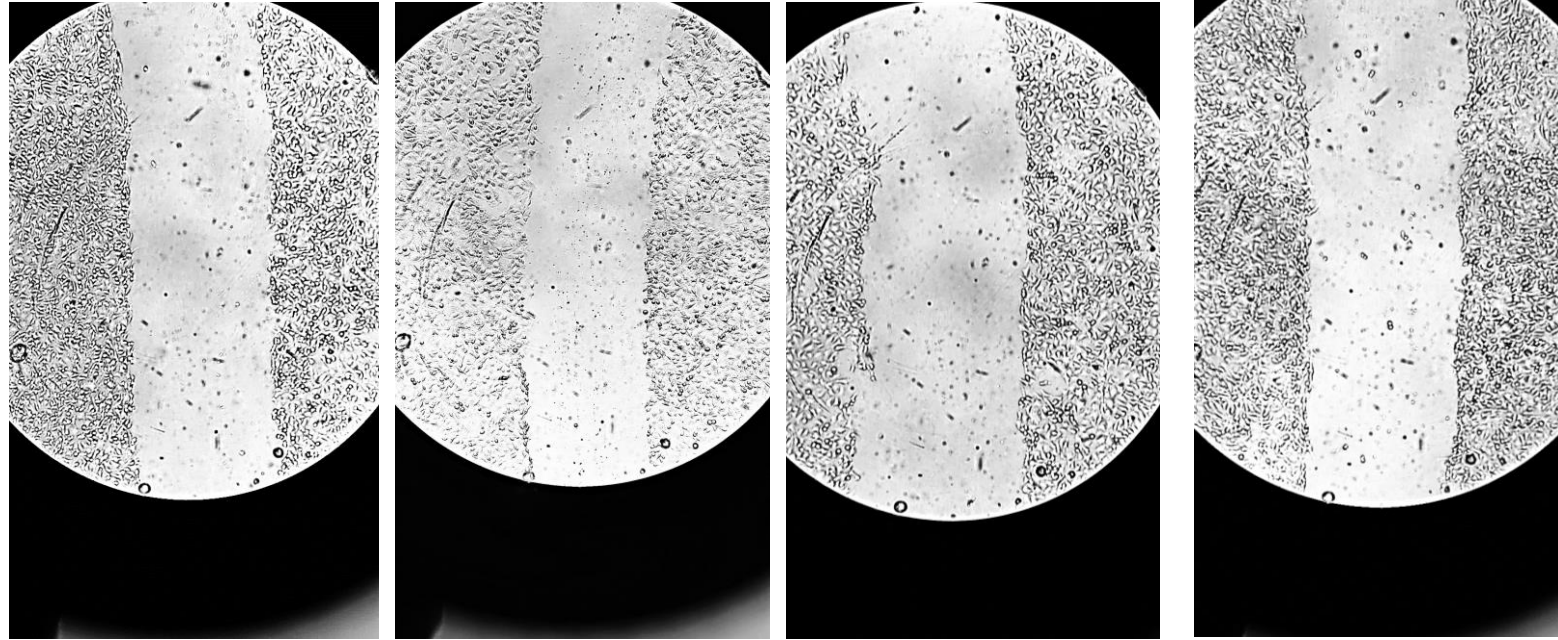

**24 h**

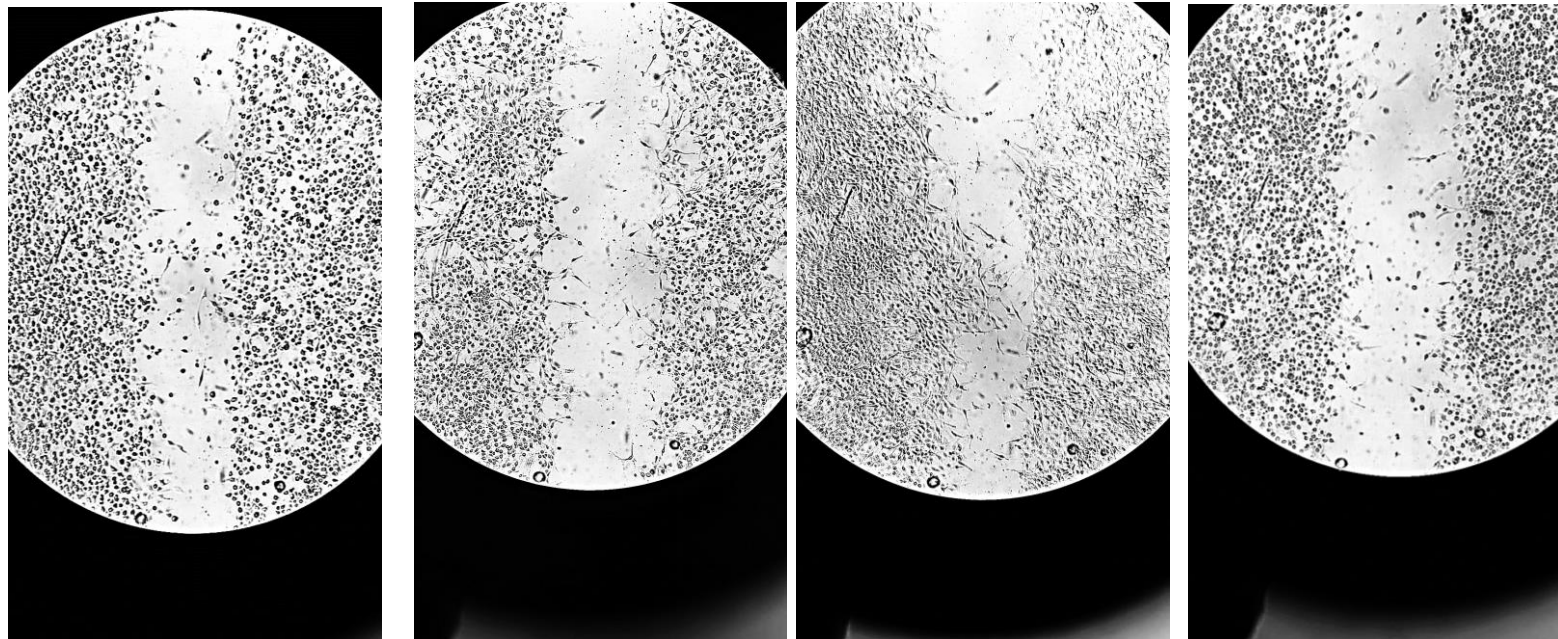

**Figure 8 C)**

**0 h**

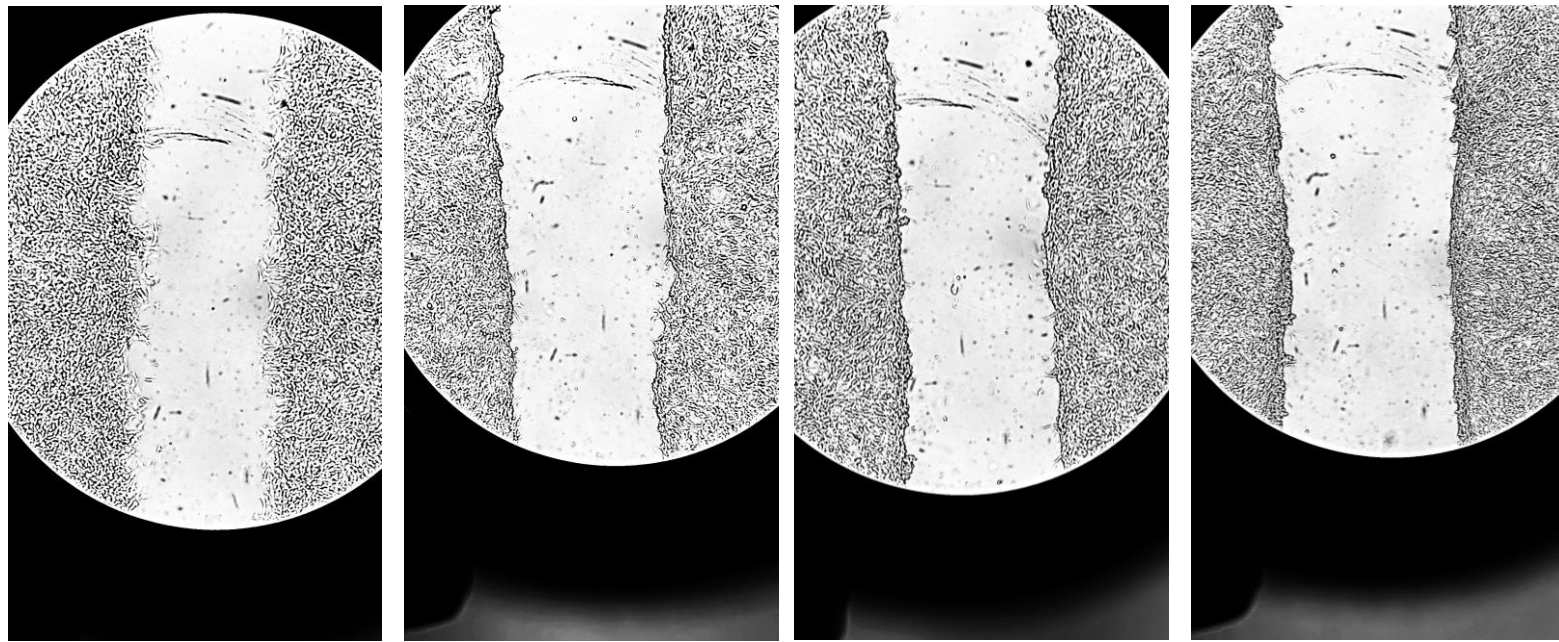

**24 h**

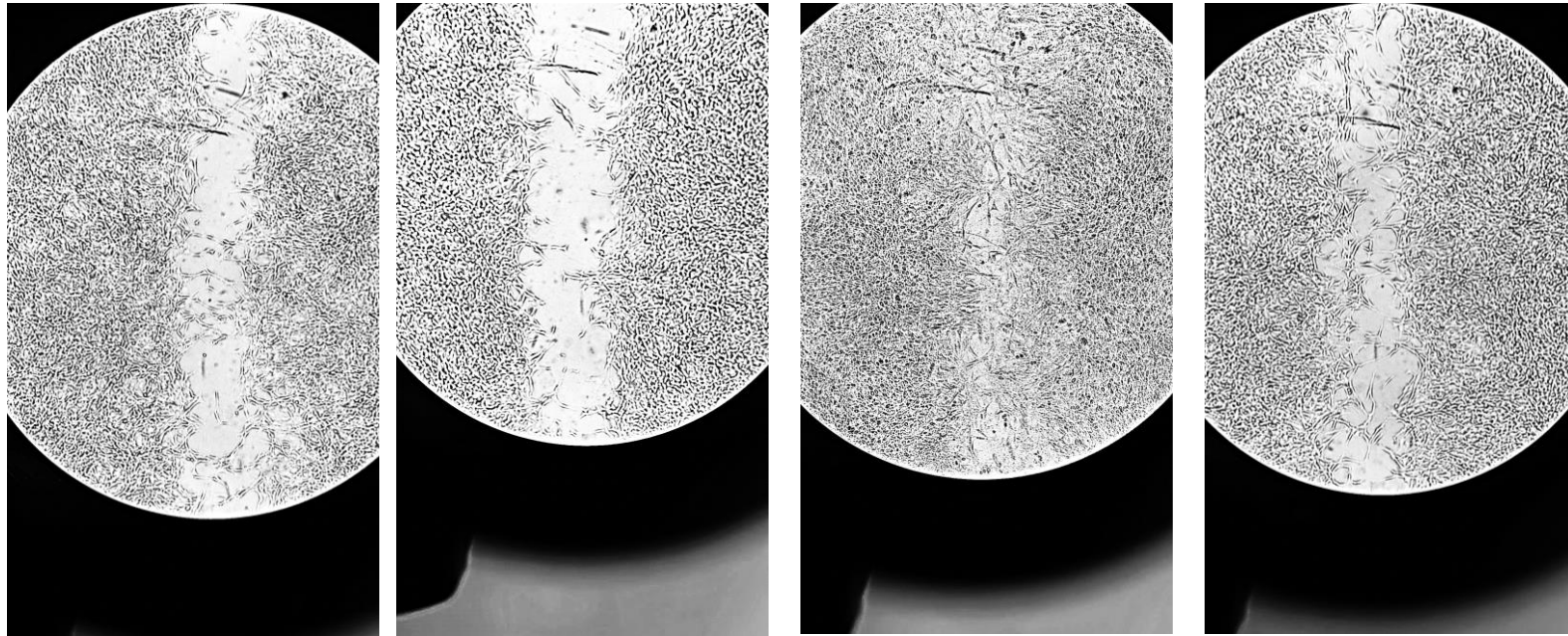

Supplement: Supplementary file 1 [file ijms-25-05389-s001.zip › Supplementary files/Supplementary file (S2).pdf]

Figure 2

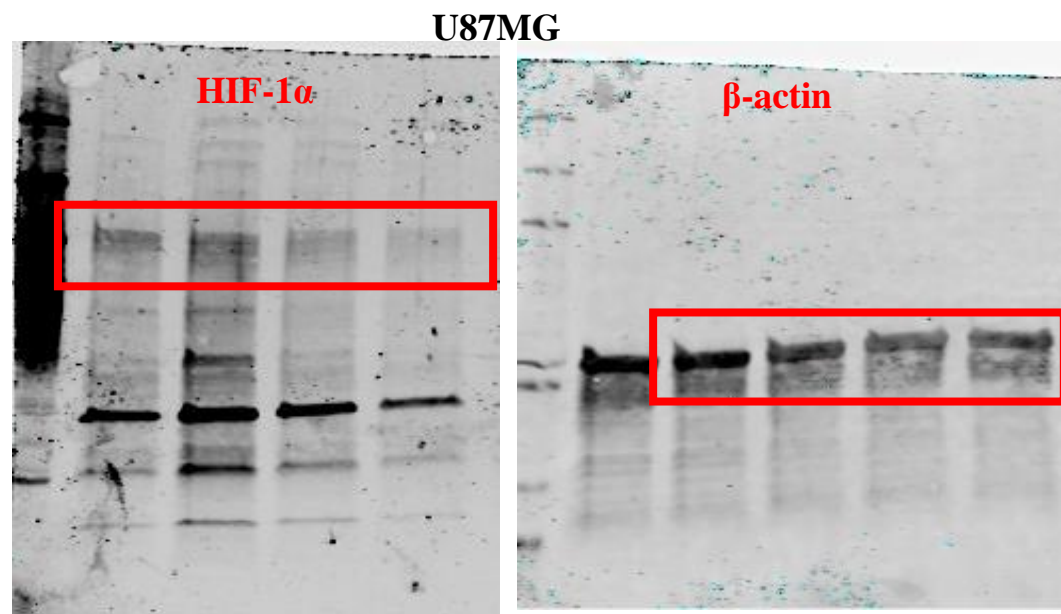

Figure 3

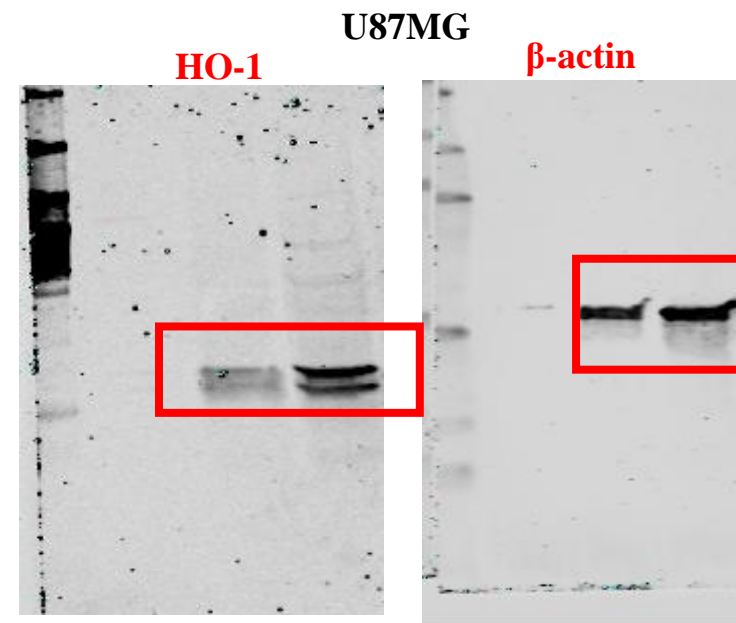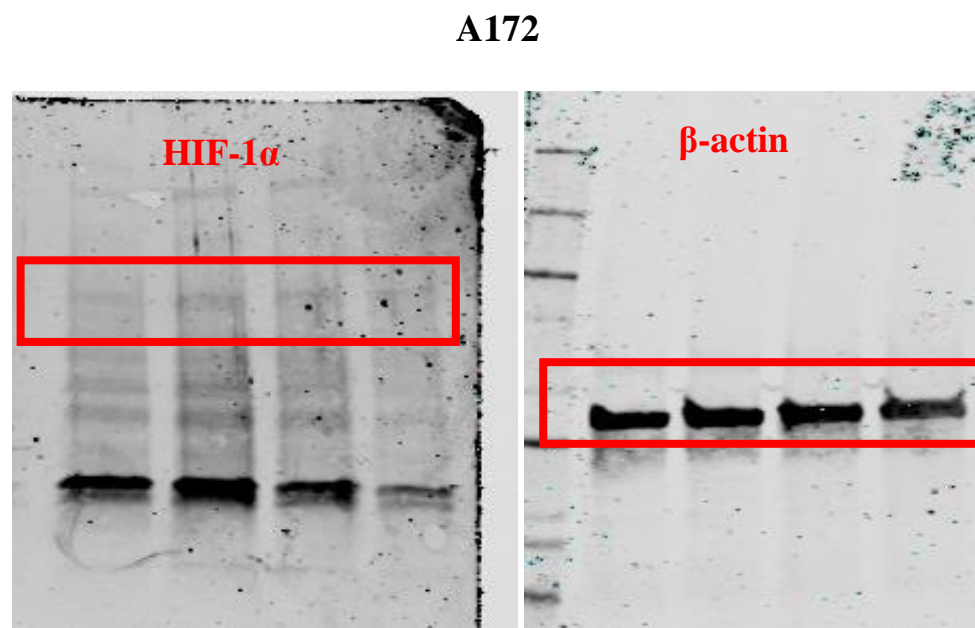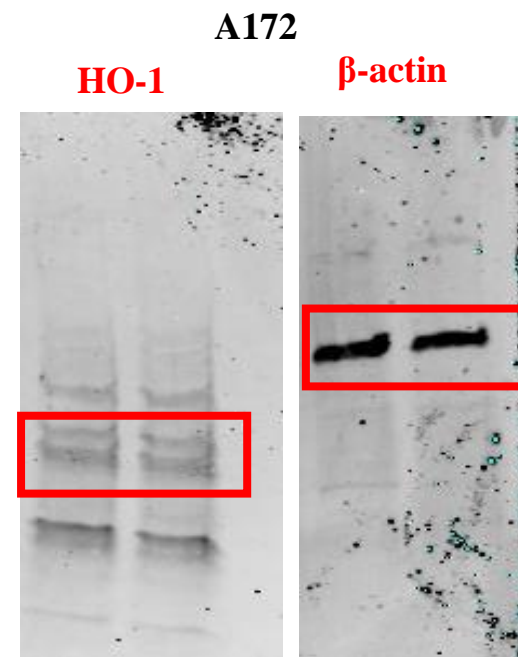

U87MG

Figure 6

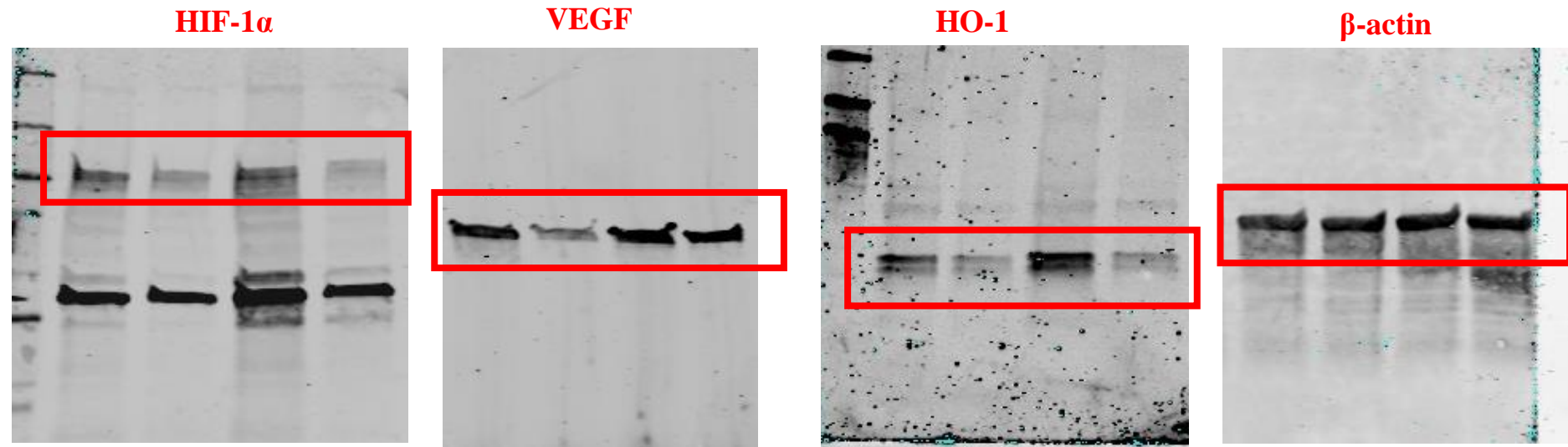

A172

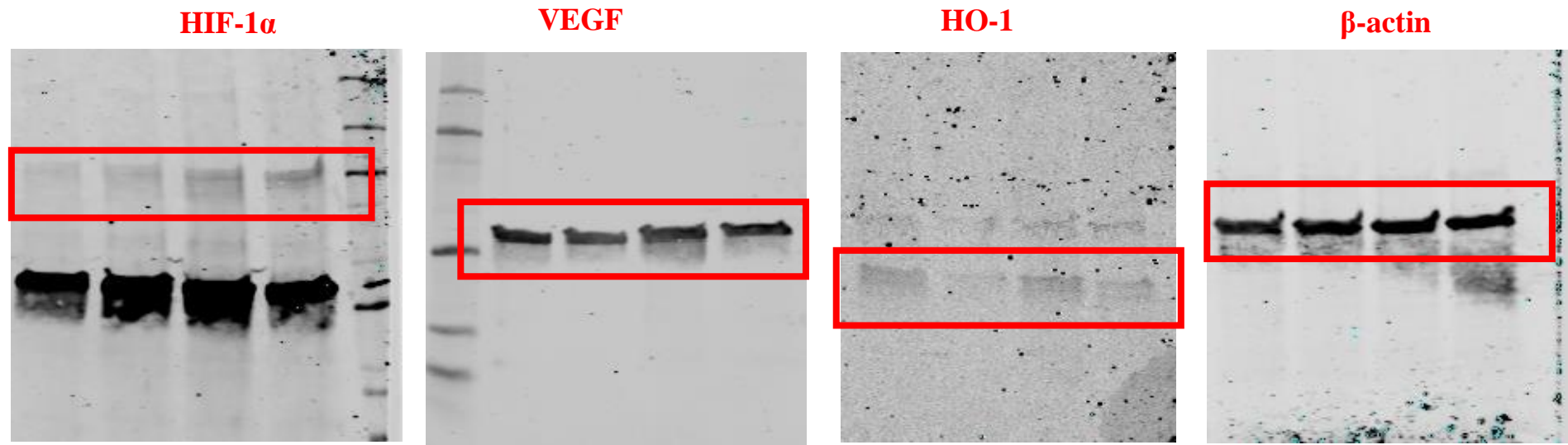

Supplement: Supplementary file 1 [file ijms-25-05389-s001.zip › Supplementary files/Supplementary file (S1).pdf]
